# The burden of incidental SARS-CoV-2 infections in hospitalized patients across pandemic waves in Canada

**DOI:** 10.1038/s41598-023-33569-2

**Published:** 2023-04-24

**Authors:** Finlay A. McAlister, Jeffrey P. Hau, Clare Atzema, Andrew D. McRae, Laurie J. Morrison, Lars Grant, Ivy Cheng, Rhonda J. Rosychuk, Corinne M. Hohl, The Canadian COVID-19 Emergency Department Rapid Response Network (CCEDRRN) Investigators, Hana Wiemer, Patrick Fok, Samuel Campbell, Kory Arsenault, Tara Dahn, Corinne DeMone, Kavish Chandra, Jacqueline Fraser, Patrick Archambault, Joel Turner, Éric Mercier, Greg Clark, Éric Mercier, Sébastien Robert, Sébastien Robert, Martyne Audet, Alexandra Nadeau, Audrey Nolet, Xiaoqing Xue, David Iannuzzi, Chantal Lanthier, Laurie Morrison, Ivy Cheng, Steven Brooks, Connie Taylor, Jeffrey Perry, Michelle Welsford, Rob Ohle, Justin Yan, Rohit Mohindra, Megan Landes, Konika Nirmalanathan, Vlad Latiu, Joanna Yeung, Natasha Clayton, Tom Chen, Jenna Nichols, Tomislav Jelic, Kate Mackenzie, Phil Davis, Aimee Goss, Andrew McRae, Brian Rowe, Katie Lin, Stephanie VandenBerg, Jake Hayward, Jaspreet Khangura, Stacey Lobos, Stacy Ruddell, Natalie Runham, Karlin Su, Corinne Hohl, Frank Scheuermeyer, Daniel Ting, Maja Stachura, Balijeet Braar, John Taylor, Ian Martin, Sean Wormsbecker, Lee Graham, Josie Kanu, Taylor Bootsma, Bernice Huynh, Amanda Swirhun, Tracy Taylor, Mai Hayashi, Mackenzie Cheyne, Neenah Williams, Katherine Lam, Kelsey Compagna, Jeffrey Hau, Vi Ho, Serena Small, Amber Cragg, Vicky Xu

**Affiliations:** 1 The Division of General Internal Medicine, Faculty of Medicine and Dentistry, University of Alberta, 5-134C Clinical Sciences Building, 11350 83 Avenue, Edmonton, AB T6G 2G3 Canada; 2 The Alberta Strategy for Patient Oriented Research Support Unit, Edmonton, Canada; 3 Department of Emergency Medicine, University of British Columbia, Vancouver, Canada; 4 Department of Emergency Services, Sunnybrook Health Sciences Centre, Toronto, ON Canada; 5 Division of Emergency Medicine, Department of Medicine, University of Toronto, Toronto, ON Canada; 6 ICES, Toronto, ON Canada; 7 Department of Emergency Medicine and Community Health Sciences, University of Calgary, Calgary, AB Canada; 8 Department of Emergency Medicine, McGill University, Montreal, QC Canada; 9 Lady Davis Institute for Medical Research, Montreal, QC Canada; 10 Department of Pediatrics, Faculty of Medicine and Dentistry, University of Alberta, Edmonton, AB Canada; 11 Emergency Department, Vancouver General Hospital, Vancouver, BC Canada; 12 Dartmouth General Hospital, Dartmouth, NS Canada; 13 Halifax Infirmary, Halifax, NS Canada; 14 Hants Community Hospital, Windsor, NS Canada; 15 Cobequid Community Health Centre, Lower Sackville, NS Canada; 16 Secondary Assessment Centers of Dartmouth General and Halifax Infirmary, Dartmouth, NS Canada; 17 Dalhousie University, Halifax, NS Canada; 18 Saint John Regional Hospital and Dalhousie University, Saint John, NS Canada; 19 Hotel-Dieu de Lévis, Lévis, QC Canada; 20 Jewish General Hospital, Montreal, QC Canada; 21 Centre Hospitalier de L’Université Laval (CHU de Québec), Quebec, QC Canada; 22 L’hôpital Royal Victoria-Royal Victoria Hospital, Montreal, QC Canada; 23 Hôpital de L’Enfant-Jésus, Quebec, QC Canada; 24 Hôpital du Saint-Sacrement, Quebec, QC Canada; 25 Hôpital Saint-François d’Assise, Quebec, QC Canada; 26 Hôtel-Dieu de Québec, CHU de Québec, Quebec, QC Canada; 27 IUCPQ: Institut Universitaire de Cardiologie et de Pneumologie de Québec, Quebec, QC Canada; 28 Hôpital du Sacré-Coeur de Montreal, Montreal, QC Canada; 29 Centre Intégré de Santé et de Services Sociaux de Chaudière-Appalaches (Hôtel-Dieu de Lévis Site), Lévis, QC Canada; 30 CHU de Québec Université Laval, Quebec City, QC Canada; 31 Centre Intégré de Santé et de Services Sociaux de Chaudière-Appalaches (Hôtel-Dieu de Lévis Site, Quebec, QC Canada; 32 Jewish General Hospital, Montréal, QC Canada; 33 McGill University Health Center, Montréal, QC Canada; 34 Hôpital du Sacré-Cœur de Montréal, Montréal, QC Canada; 35 Sunnybrook, Toronto, ON Canada; 36 Queens University, Kingston, ON Canada; 37 The Ottawa Hospital, Ottawa, ON Canada; 38 Hamilton General Hospital, Hamilton, ON Canada; 39 Health Science North, Sudbury Ontario, ON Canada; 40 University Hospital and Victoria Hospital-London Health Sciences Centre, London, ON Canada; 41 North York General Hospital, Toronto, ON Canada; 42 Toronto Western Hospital, Toronto, ON Canada; 43 University Health Network, Toronto, ON Canada; 44 Kingston General Hospital, Hotel Dieu Hospital, Kingston, ON Canada; 45 Sunnybrook Health Sciences Center, Toronto, ON Canada; 46 Hamilton General Hospital, Juravinski Hospital, Hamilton, ON Canada; 47 London Health Sciences Centre, London, ON Canada; 48 Health Sciences North, Sudbury, ON Canada; 49 Health Sciences Centre, Winnipeg, MB Canada; 50 St Paul’s Hospital, Royal University Hospital, Saskatoon City Hospital, Saskatoon, SK Canada; 51 University of Saskatchewan, Saskatoon, SK Canada; 52 Peter Lougheed Centre and Rockyview, Calgary, AB Canada; 53 University of Alberta Hospital, Edmonton, AB Canada; 54 Foothills, Calgary, AB Canada; 55 South Campus, Calgary, AB Canada; 56 Northeast Community Health Centre and Royal Alexandra Hospital, Edmonton, AB Canada; 57 University of Calgary, Calgary, AB Canada; 58 Foothills Medical Centre, Peter Lougheed Centre, Rockyview General Hospital, South Health Campus, Calgary, AB Canada; 59 Royal Alexandra Hospital/Northeast Community Health Center, Edmonton, AB Canada; 60 Saint Paul’s Hospital and Mount St Joseph’s, Vancouver, BC Canada; 61 Vancouver General Hospital, Vancouver, BC Canada; 62 Lions Gate Hospital, North Vancouver, BC Canada; 63 Surrey Memorial Hospital, Surrey, BC Canada; 64 Royal Columbian Hospital, New Westminster, BC Canada; 65 Abbotsford Regional Hospital and Royal Inland Hospital, Abbotsford, BC Canada; 66 Eagle Ridge Hospital, Port Moody, BC Canada; 67 Kelowna General Hospital, Kelowna, BC Canada; 68 University of British Columbia, Vancouver, BC Canada; 69 St. Paul’s Hospital, Mount Saint Joseph, Vancouver, BC Canada; 70 Abbotsford Regional Hospital and Cancer Center, Abbotsford, BC Canada; 71 Royal Columbian Hospital, New Westminster, BC Canada; 72 Eagle Ridge Hospital and Health Care Centre, Port Moody, BC Canada; 73 Royal Inland Hospital, Kamloops, BC Canada; 74 Lions Gate Hospital, Vancouver, BC Canada; 75 University of British Columbia Support Staff, Vancouver, BC Canada

**Keywords:** SARS-CoV-2, Health services

## Abstract

Many health authorities differentiate hospitalizations in patients infected with SARS-CoV-2 as being “for COVID-19” (due to direct manifestations of SARS-CoV-2 infection) versus being an “incidental” finding in someone admitted for an unrelated condition. We conducted a retrospective cohort study of all SARS-CoV-2 infected patients hospitalized via 47 Canadian emergency departments, March 2020-July 2022 to determine whether hospitalizations with “incidental” SARS-CoV-2 infection are less of a burden to patients and the healthcare system. Using a priori standardized definitions applied to hospital discharge diagnoses in 14,290 patients, we characterized COVID-19 as (i) the “Direct” cause for the hospitalization (70%), (ii) a potential “Contributing” factor for the hospitalization (4%), or (iii) an “Incidental” finding that did not influence the need for admission (26%). The proportion of incidental SARS-CoV-2 infections rose from 10% in Wave 1 to 41% during the Omicron wave. Patients with COVID-19 as the direct cause of hospitalization exhibited significantly longer LOS (mean 13.8 versus 12.1 days), were more likely to require critical care (22% versus 11%), receive COVID-19-specific therapies (55% versus 19%), and die (17% versus 9%) compared to patients with Incidental SARS-CoV-2 infections. However, patients hospitalized with incidental SARS-CoV-2 infection still exhibited substantial morbidity/mortality and hospital resource use.

## Introduction

The number of patients hospitalized with SARS-CoV-2 infection remains high in this third year of the pandemic. Our understanding of the impact of these hospitalizations on the healthcare system has been clouded by debate about whether we should include only those hospitalizations that are “for COVID-19” (i.e., direct manifestations of SARS-CoV-2 infection) or also those admitted for an unrelated condition and incidentally found to have SARS-CoV-2 infection^1–4^.

There is no standardized definition, however, to determine which hospitalized patients infected with SARS-CoV-2 were admitted “for COVID-19” and which have an incidental infection. In Canada and the United Kingdom, hospitals use ad hoc categorizations created by local infection prevention and control teams to classify admissions, with variability between institutions on whether provision of COVID-specific therapies such as remdesivir or tocilizumab are included in these definitions^5,6^. In the United States, the Centers for Disease Control defines a hospitalization as being “for COVID-19” if ICD-10 code U07.1 was (i) the primary discharge diagnosis or (ii) a secondary diagnosis and the patient was treated with remdesivir or had a primary diagnosis of sepsis, pulmonary embolism, acute respiratory failure, or pneumonia^7^. On the other hand, some US States only define hospitalizations as being “for COVID-19” if COVID-19 was the primary or secondary diagnosis and dexamethasone was prescribed^8^. However, a recent study of electronic health records (EHR) from 4 US health care systems found that EHR phenotypes that include discharge diagnosis codes were the best means to classify the cause of admissions, as laboratory tests or treatments for patients with COVID-19 were not standardized across systems or even across hospitals within the same system^9^. Without a standard definition that accurately differentiates the truly “incidental” infection (an asymptomatic patient not requiring treatment) from all other SARS-CoV-2 positive patients, the healthcare needs of some hospitalized patients will be misjudged, leading to poor future resource allocation and contributing to system-wide capacity breakdowns.

In this study, we describe the proportion of patients with SARS-CoV-2 infection hospitalized during the various pandemic waves in Canada in whom COVID-19 was adjudged (using a priori standardized definitions derived by consensus of clinicians in emergency medicine, infectious diseases, and general internal medicine and based on hospital discharge diagnoses) to be (i) the Direct cause for the admission, versus (ii) a potential Contributing factor for the admission, versus (iii) an Incidental finding, as well as their resource use and outcomes.

## Methods

### Study design, setting, participants, and data sources

As described in full elsewhere^10^ and on our website (www.ccedrrn.com) data for the Canadian COVID-19 Emergency Department Rapid Response Network (CCEDRRN) population-based registry were retrospectively collected via manual chart review from 47 emergency departments (EDs), encompassing a mix of academic and community-based EDs, located in 6 provinces across Canada. We included all patients hospitalized between March 1/20 and July 24/22 who had confirmed SARS-CoV-2 infection using a nucleic acid amplification test or rapid antigen test in the 14 days prior to or during their index ED visit, or in the first 5 days after hospitalization, confirmed positive at a healthcare facility. Thus, our focus was on patients admitted with SARS-CoV-2 infection rather than nosocomially acquired infections. Trained research assistants at each site collected information on baseline characteristics, hospital course, treatments, and outcomes within 30 days. For patients with multiple hospitalizations and/or positive tests during the study period, we included only their first hospitalization related to a positive test within 14 days. As described below, we used a priori standardized definitions to classify the cause of hospitalization on the basis of the hospital (not ED) discharge diagnoses.

### Variable definitions

Prior work from our group demonstrated that it is challenging to accurately dichotomize hospitalizations into only 2 groups (those that are “for COVID-19” versus “with incidental infection”) using discharge diagnoses data^11^. As the data collected in the study case report forms included the most responsible diagnosis at the time of discharge and up to 10 secondary diagnoses but not the background rationale, symptom timelines, or supporting notes, we chose to instead classify hospitalizations into one of 3 categories (adapted from the Centres for Disease Control classification system and based on definitions established by expert consensus of the ED physicians and hospital internists in the CCEDRRN)^7^. We used the most responsible diagnosis assigned at hospital discharge to classify each hospitalization in a patient with SARS-CoV-2 infection at the time of admission as:

1. COVID-19 is the direct cause of admission (herewith referred to as the “Direct” group) if the Most Responsible Diagnosis at discharge was any of the following: COVID-19, upper respiratory tract infection, pneumonia, viral pneumonia, sinusitis, pharyngitis, bronchitis, flu-like illness, Adult Respiratory Distress Syndrome, respiratory failure, or sepsis.
2. COVID-19 is a potential contributing factor for the hospitalization (herewith referred to as the “Contributing” group) if the Most Responsible Diagnosis at discharge was:
  thromboembolic phenomena (including acute coronary syndrome, ischemic stroke, transient ischemic attack, other arterial clot, pulmonary embolism, or deep venous thrombosis),
  acute kidney injury,
  electrolyte abnormalities (hypo- or hypernatremia, hypo- or hyperkalemia, hypo- or hypercalcemia, hypo- or hypermagnesemia)
  exacerbations of heart failure, asthma, COPD, or Multiple Sclerosis
  Delirium, Confusion, Altered level of consciousness
  Diabetic ketoacidosis
  Syncope or falls,
  Acute functional decline,
  Rhabdomyolysis,
  Mental health issues (anxiety, depression)
  Overdose/suicide attempt
  Chest pain or angina (stable or unstable), or ischemic heart disease
3. SARS-CoV-2 infection is an incidental finding if the Most Responsible Diagnosis at discharge was anything other than those listed under 1 or 2 above (*herewith referred to as the “Incidental” group*). Note that this is not synonymous with the patient being asymptomatic.

We defined the Canadian pandemic waves as: Mar 1/20–June 30/20 (wave 1), July 1/20–Feb 14/21 (wave 2), Feb 15/21–Jul 14/21 (wave 3, driven largely by the spread of the alpha variant across Canada), July 15/21–Nov 27/21 (wave 4, driven largely by the spread of the delta variant across Canada), and Nov 28/21 onwards (wave 5, beginning when the first case of omicron was reported in Canada).

### Statistical analyses

Summary statistics (e.g., mean, standard deviation [SD], median, interquartile range, count, percent) describe the patient characteristics, acute care utilization, and outcomes. Differences among hospitalization groups were assessed by chi-square tests and analysis of variance. To ensure patient privacy, a cell size restriction policy prohibited us from reporting counts of less than six. A p-value < 0.05 was considered statistically significant. All analyses were performed in Stata (Version 16.1, StataCorp, College Station, Texas).

### Ethics approval

The University of British Columbia (UBC) Clinical Research Ethics Board reviewed and approved the full study protocol with a waiver for individual patient informed consent given its observational nature, allowing us to capture consecutive patient samples in participating EDs. Each participating ED obtained consent for registry data collection from their local Institutional Ethics Review Boards as well. Our work was performed in accordance with the Declaration of Helsinki.

## Results

**Figure 1:**
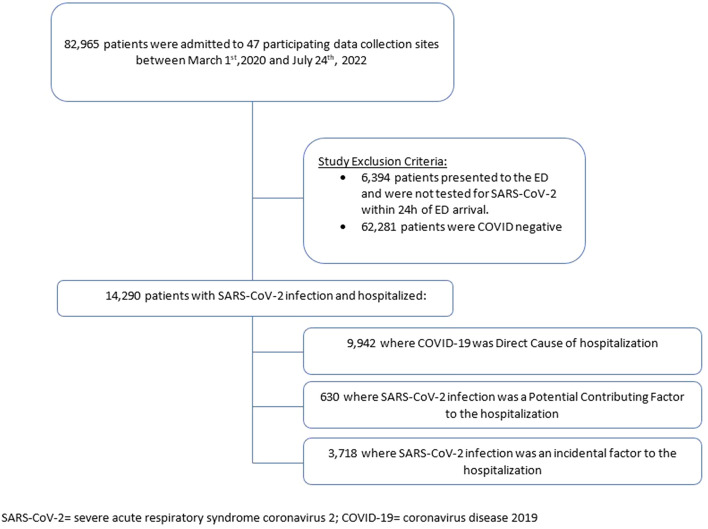
Participant flow diagram.

Of the 82,965 patients presenting to the 47 participating EDs during the study, 14,290 (17%) had confirmed SARS-CoV-2 infection based on PCR testing, and were admitted to hospital (Fig. 1): 9942 (70%), 630 (4%) and 3718 (26%) were adjudged to have COVID-19 as the Direct cause, a potential Contributing factor, or an Incidental finding for their hospitalization, respectively.

While COVID-associated symptoms were more common in those with hospitalizations deemed to be Directly due to COVID-19 (96%), 82% of the Contributing group and 75% of the Incidental group exhibited at least one COVID-related symptom on presentation to the ED (Table 1). The most common were shortness of breath (50%), cough (45%), generalized weakness (37%), or fever/chills (32%). Patients with Incidental SARS-CoV-2 infection were younger and had less hypertension, diabetes, underlying lung disease, or moderate or severe liver disease, but were more likely to have active cancer, and to smoke or misuse alcohol or illicit substances, compared to those patients hospitalized for COVID-19 (Table 1). Patients hospitalized for COVID-19 were more likely to present with Canadian Triage and Acuity Scale (CTAS) status 1 or 2 (emergent or needing acute resuscitation) at presentation. Among those with known vaccine status, hospitalizations in unvaccinated patients were more likely to be Direct COVID-19 admissions (75%) than hospitalizations in fully vaccinated patients (53%, p < 0.001).

**Table 1:** Baseline characteristics stratified by type of admission in 14,290 patients with SARS-CoV-2 infection.

	COVID-19 is direct cause of hospitalization (n = 9942)	COVID-19 is a potential contributing factor for hospitalization (n = 630)	SARS-CoV-2 infection is incidental and not related to cause of hospitalization (n = 3718)	All SARS-CoV-2 infected patients (n = 14,290)	P-value comparing across 3 categories
Age (in years) median (IQR)	70 (24)	73 (27)	63 (36)	69 (28)	< 0.001
Female (%)	4251 (42.8)	291 (46.2)	1645 (44.2)	6187 (43.3)	0.24
Institutionalized (%)	1076 (10.8)	62 (9.8)	401 (10.8)	1539 (10.8)	0.74
Goals of care (%)					
Full ventilator resuscitation, no CPR	15 (0.2)	< 5	8 (0.2)	26 (0.2)	< 0.01
Full code	9024 (90.9)	562 (89.2)	3465 (93.2)	13,061 (91.4)	
Do not resuscitate	410 (4.1)	21 (3.3)	107 (2.9)	549 (3.8)	
Comorbidities (%)					
Hypertension	4807 (48.4)	314 (49.8)	1434 (38.6)	6555 (45.9)	< 0.001
Diabetes	2630 (26.5)	179 (28.4)	747 (20.1)	3556 (24.9)	< 0.001
Coronary artery disease	1327 (13.4)	84 (13.3)	441 (11.9)	1852 (13.0)	0.07
Heart failure	782 (7.9)	45 (7.1)	321 (8.6)	1148 (8.0)	0.24
Chronic kidney disease, no dialysis	1000 (10.1)	76 (12.1)	376 (10.1)	1452 (10.2)	0.27
Dialysis	93 (0.9)	< 5	46 (1.2)	141 (1.0)	0.06
Organ transplant	195 (2.0)	10 (1.6)	45 (1.2)	250 (1.6)	< 0.05
Rheumatologic disorder	1043 (10.5)	66 (10.5)	349 (9.4)	1458 (10.2)	0.16
Asthma	713 (7.2)	25 (4.0)	212 (5.7)	950 (6.7)	< 0.001
Chronic lung disease, including COPD or pulmonary fibrosis but not asthma	1247 (12.5)	40 (6.4)	342 (9.2)	1629 (11.4)	< 0.001
Active cancer	775 (7.8)	44 (7.0)	339 (9.1)	1158 (8.1)	< 0.05
Moderate/severe liver disease	417 (4.2)	12 (1.9)	78 (2.1)	507 (3.6)	< 0.001
Tobacco use, (%)	625 (6.3)	49 (7.8)	423 (11.4)	1097 (7.7)	< 0.001
Alcohol misuse, (%)	370 (3.7)	40 (6.4)	335 (9.0)	745 (5.2)	< 0.001
Illicit substance use, (%)	348 (3.5)	71 (11.3)	389 (10.5)	808 (5.6)	< 0.001
Arrival by ambulance, (%)	6631 (66.7)	413 (65.6)	2162 (58.2)	9206 (64.4)	< 0.001
Canadian Triage Acuity Score, (%)					
CTAS 1 (resuscitation)	1051 (10.6)	33 (5.2)	335 (9.0)	1419 (9.9)	< 0.001
CTAS 2 (emergent)	4513 (45.4)	281 (44.6)	1489 (40.0)	6283 (44.0)	
CTAS 3 (urgent)	3822 (38.4)	283 (44.9)	1654 (44.5)	5759 (40.3)	
CTAS 4 (less urgent)	509 (5.1)	32 (5.1)	207 (5.6)	748 (5.2)	
CTAS 5 (non-urgent)	31 (0.3)	< 5	25 (0.7)	57 (0.4)	
Symptoms reported at ED arrival, (%)					
Cough	5493 (55.3)	141 (22.4)	755 (20.3)	6389 (44.7)	< 0.001
Dyspnea	6130 (61.7)	149 (23.7)	914 (24.6)	7193 (50.3)	< 0.001
Fever and/or chills	3968 (39.9)	69 (11.0)	576 (15.5)	4613 (32.3)	< 0.001
General weakness	4187 (42.1)	205 (32.5)	952 (25.6)	5344 (37.4)	< 0.001
Chest pain	1809 (18.2)	127 (20.2)	603 (16.2)	2539 (17.8)	< 0.01
Abdominal pain	801 (8.1)	65 (10.3)	789 (21.2)	1655 (11.6)	< 0.001
Diarrhea	1591 (16.0)	54 (8.6)	346 (9.3)	1991 (13.9)	< 0.001
Nausea/vomiting	1812 (18.2)	126 (20.0)	869 (23.4)	2807 (19.6)	< 0.001
Headache	973 (9.8)	49 (7.8)	274 (7.4)	1296 (9.1)	< 0.001
Rhinorrhea	402 (4.0)	11 (1.8)	66 (1.8)	479 (3.4)	< 0.001
Myalgia/Arthralgia	1034 (10.4)	46 (7.3)	224 (6.0)	1304 (9.1)	< 0.001
Sore throat	794 (8.0)	28 (4.4)	135 (3.6)	957 (6.7)	< 0.001
Altered mental status	1760 (17.7)	261 (41.4)	751 (20.2)	2772 (19.4)	< 0.001
Dysgeusia/anosmia	350 (3.5)	8 (1.3)	27 (0.7)	385 (2.7)	< 0.001
Any COVID related symptom, (%)	9525 (95.8)	516 (81.9)	2795 (75.2)	12,836 (89.8)	< 0.001
Vaccination status at time of the ED visit, (%)					
Known unvaccinated	6190 (62.3)	320 (50.8)	1738 (46.7)	8248 (57.7)	< 0.001
Partially vaccinated	275 (2.8)	20 (3.2)	86 (2.3)	381 (2.7)	
Fully vaccinated	557 (5.6)	86 (13.7)	400 (10.8)	1043 (7.3)	
Unknown vaccination status	2920 (29.4)	204 (32.4)	1494 (40.2)	4618 (32.3)	

*Likely an under-count as not collected routinely until Feb, 2021.

While any of the COVID therapies (dexamethasone, remdesivir, tocilizumab, sotrovimab, or nirmatrelvir/ritonavir) were more likely to have been dispensed to the Direct Hospitalization group (55%), they were also prescribed in 21% of the Contributing group and in 19% of patients in the Incidental group (Table 2).

**Table 2:** Acute care utilization and outcomes, by most responsible diagnosis.

	COVID-19 is direct cause of hospitalization (n = 9942)	COVID-19 is a potential contributing factor for hospitalization (n = 630)	SARS-CoV-2 infection is incidental and not related to cause of hospitalization (n = 3,718)	P-value comparing across 3 categories
Admissions, (%)				
One admission	9373 (94.3)	597 (94.8)	3515 (94.5)	0.39
Two admissions	496 (5.0)	32 (5.1)	172 (4.6)	
Three or more admissions	73 (0.7)	< 5	31 (0.8)	
Hospital length of stay, per admission, mean (SD)	13.8 (19.9)	12.4 (15.0)	12.1 (16.1)	< 0.001
Hospital length of stay, per admission, median (IQR)	8 (12)	7 (11)	7 (11)	< 0.001
Admitted to critical care during index admission, (%)	2181 (21.9)	59 (9.4)	423 (11.4)	< 0.001
Critical care days for those admitted to critical care during index admission, mean (SD)	14.7 (25.2)	5.6 (6.7)	9.5 (14.9)	< 0.001
Critical care days for those admitted to critical care during index admission, median (IQR)	9 (13)	2.5 (7)	4 (8)	< 0.001
Supplemental oxygen in ED, (%)	4296 (43.2)	101 (16.0)	540 (14.5)	< 0.001
Most aggressive form of oxygen delivery used during index admission, (%)				
Mechanical ventilation	1153 (11.6)	21 (3.3)	200 (5.4)	< 0.001
CPAP/BiPAP	142 (1.4)	< 5	29 (0.8)	
High-flow nasal oxygen	559 (5.6)	7 (1.1)	38 (1.0)	
Simple or non-rebreather facemask	1037 (10.4)	16 (2.5)	128 (3.4)	
Nasal prongs	3511 (35.3)	87 (13.8)	432 (11.6)	
COVID-19 therapies used during index admission, (%)				
Dexamethasone	5223 (52.5)	113 (17.9)	665 (17.9)	< 0.001
Remdesivir*	523 (5.3)	10 (1.6)	45 (1.2)	< 0.001
Tocilizumab*	68 (0.7)	< 5	< 5	< 0.001
Sotrovimab*	48 (0.5)	11 (1.8)	16 (0.4)	< 0.001
Nirmatrelvir/Ritonavir	11 (0.1)	< 5	8 (0.2)	0.31
Any COVID-19 therapy during index admission	5430 (54.6)	130 (20.6)	700 (18.8)	< 0.001
Death within 30 days, (%)	1637 (16.5)	35 (5.6)	337 (9.1)	< 0.001

*ED* Emergency Department, *SD *standard deviation, *CPAP* Continuous positive airway pressure, *BiPAP* Bilevel airway pressure.

*Likely an under-count as not collected routinely until Feb, 2021.

**Figure 2:**
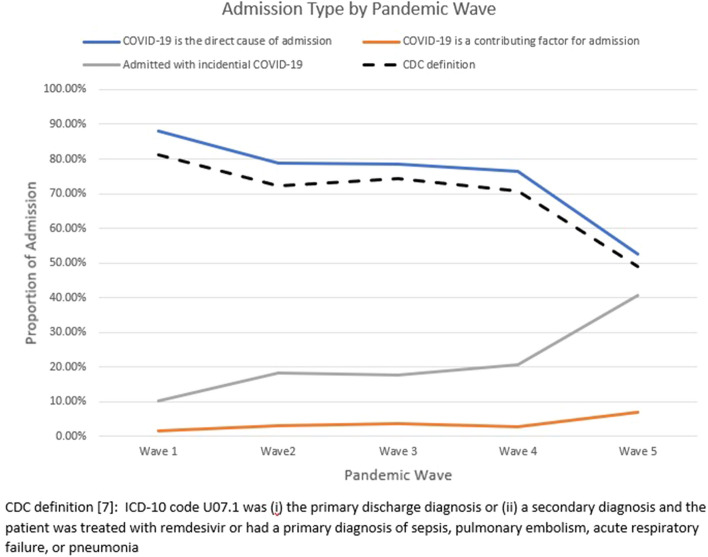
Admission type by pandemic wave in study cohort.

While COVID-19 was deemed the Direct cause of the admission in 88% of patients hospitalized during the first pandemic wave, this decreased markedly (Fig. 2) to 53% of hospitalizations during wave 5 (corresponding to the Omicron surge in Canada). Of note, 99% of hospitalizations where COVID-19 was deemed to be the Direct cause had a most responsible diagnosis of COVID-19 with respiratory manifestations. The proportion of SARS-CoV-2 positive hospitalizations that were deemed to be directly due to COVID-19 using our criteria was similar across provinces in each wave (eFigure 1) and was consistently larger than the proportion of hospitalizations meeting the CDC definition for a hospitalization “caused by COVID-19” (Fig. 2). Of the 8246 hospitalizations in known unvaccinated patients, 6190 (75.1%) were for COVID-19 directly and of the 381 hospitalizations in partially vaccinated patients, 275 (72.1%); in comparison, in fully vaccinated patients 557 (53.4%) of 1043 hospitalizations were for COVID-19 (Table 1).

SARS-CoV-2 was a potential contributing factor in 630 hospitalizations (4% overall, ranging from 2% in Wave 1 to 7% in the Omicron wave). The most common primary diagnoses (e Appendix Table e1) in the Contributing group were diseases of the nervous system (such as stroke or delirium), poisonings or injuries (including adverse drug reactions, alcohol misuse, and falls), endocrine or metabolic abnormalities (hyponatremia, diabetic ketoacidosis, or other electrolyte disturbances), or circulatory system conditions (principally heart failure, arrhythmias, or acute coronary syndrome). SARS-CoV-2 was deemed Incidental in 3718 admissions (26% overall, ranging from 10% in Wave 1–41% in the Omicron wave). The most common primary diagnoses in the Incidental group were diseases of the digestive system (abdominal surgeries, liver disorders, diverticulitis or cholecystitis, and bowel obstructions), non-cardiac circulatory system conditions (principally hypertension), or non-stroke non-delirium diseases of the nervous system (principally seizures, dementia, or lower motor neuron conditions).

Patients in the Direct group exhibited significantly longer LOS and a higher rate of critical care admission (with longer critical care stays), and were more likely to die in hospital, compared to the other groups (Table 2). However, those in the Incidental group exhibited substantial morbidity: mean hospital LOS 12 days, 11% were admitted to a critical care unit (for a mean of 9.5 days), and 9% died within 30 days (Table 2). Those in the Contributing group had comparatively better outcomes, although their CTAS distribution suggests they were less sick at baseline and their mix of diagnoses was substantially different from the other categories (Table 1).

## Interpretation

Our study found that the proportion of hospitalized patients deemed to have Incidental SARS-CoV-2 infection increased proportionally across waves, but these patients still experienced substantial morbidity and mortality. We found that 75% of patients meeting our definition of “incidental infections” exhibited at least one symptom potentially attributable to COVID-19 when they presented for care, albeit that symptom may have been due to other reasons, and almost a fifth were treated with COVID-19 therapies by their attending physicians. Of note, the assignment of whether an infection was incidental rather than the cause or a contributor to the need for admission was made by our team based on the final diagnoses assigned by each patient’s attending physician at discharge, and it is thus not surprising that some patients received COVID-19 therapies during their hospital stay before the results of all tests were back and final diagnoses were apparent. In addition, it is likely that even in those who were asymptomatic and didn’t receive COVID-19 therapies, concomitant SARS-CoV-2 infection likely influenced treatment decisions and negatively impacted the length of stay for their most responsible diagnosis due to slower access to diagnostics, procedures, and allied health support due to infection control precautions. The mean hospital LOS for patients with “incidental” SARS-CoV-2 infection was 12 days, compared to an average hospital LOS in Canada of 6.9 days pre-pandemic^16^. If health authorities choose not to include “incidental” COVID-19 hospitalizations in planning, they may be substantially underestimating the current resource needs of hospitals, which could contribute to under-resourced and potentially overwhelmed hospitals. Incorporating the needs of these resource-intensive so-called ”Incidental” infections is necessary for accurate inpatient resource planning, and to direct public health measures aimed at protecting healthcare system capacity.

The diagnoses, demographics, and comorbidity profiles differed between the Direct, Contributing, and Incidental groups, therefore we cannot determine the excess mortality and morbidity that was directly attributable to SARS-CoV-2 infection in patients assigned other most responsible diagnoses. Future work would need to directly compare patients with “incidental SARS-CoV-2 infection” with a matched cohort with the same primary diagnoses but negative SARS-CoV-2 tests (this is not possible in our study as data was only systematically collected on patients with positive SARS-CoV-2 tests). However, others have shown that concomitant SARS-CoV-2 infection is associated with worse outcomes in patients with a wide variety of diagnoses^17–19^, supporting our assertion that healthcare capacity is influenced by all SARS-CoV-2 infected patients and not just those admitted specifically for COVID-19.

Our finding that the proportion of hospitalized patients with SARS-CoV-2 infection who were admitted for COVID-19 declined as the pandemic evolved is not surprising, and likely multifactorial. First, different variants of concern (with differences in transmissibility and virulence) drove each of the waves in Canada, and population prevalence rates increased with successive waves, increasing the chance that individuals hospitalized for other reasons would also have concomitant SARS-CoV-2 infection^12,13^. Second, the introduction of vaccinations and outpatient therapies also influenced clinical manifestations, severity of disease, and need for hospitalization in those with COVID-19^13^. However, although unvaccinated patients were more likely to be admitted for COVID-19 (75% of their hospitalizations with SARS-CoV-2 infection), even in fully vaccinated patients over half of hospitalizations were for COVID-19 and thus the upswing in “Incidental” SARS-CoV-2 infections over time was not just a manifestation of increasing vaccination rates. Third, as clinicians’ knowledge about the manifestations of COVID-19 expanded over time, it seems likely that their assignment of most responsible diagnoses in infected patients would have evolved commensurate with that knowledge. Our data is consistent with a recent CDC report on American hospitals that found that the proportion of admissions in SARS-CoV-2 infected patients that were attributed directly to COVID-19 declined from 84% in wave 1 to approximately half during the omicron wave^7^. Other reports have also confirmed that during the Omicron wave nearly half of hospitalizations in SARS-CoV-2 infected patients had incidental infections^9,14^. This aligns with data showing that patients infected with the Omicron variant experienced less involvement of the lower respiratory tract, less delirium, and less need for admission than those infected with earlier variants^15^.

### Limitations

As with any retrospective observational study using chart reviews, there are limitations to our work, principally around the possibility of misclassification bias. As acknowledged earlier, our study design does not let us determine causation for the poor outcomes we observed in the Incidental infection group. It should be recognized that the Incidental group was heterogeneous and included a number of patients undergoing surgery or with active cancer or substance use disorders as well as patients with less severe illnesses. However, it remains a fact that these patients used substantial in-hospital resources, and had high in-hospital mortality, which should bear on resource planning.

Unfortunately, there is no standard definition of hospitalization “for COVID-19” versus “with incidental SARS-CoV-2 infection”, with varying definitions used by regions and countries. We chose to use consensus-based definitions derived by clinicians experienced in COVID-19 care based on the most responsible diagnosis assigned at hospital discharge to classify hospitalizations, as we contend that the clinician actually caring for the patient during the course of their hospitalization is best situated to make this determination. This resulted in a higher proportion of admissions in each wave (approximately 10%) being classified as “Directly” due to COVID-19 using our definitions than would have been the case if we’d used the CDC definition (Fig. 2). In an earlier study conducted in British Columbia by our team, multiple reviewers assessed the medical charts of 1651 patients hospitalized during the omicron wave with a very high degree of consistency (kappa 0.89, 95% CI 0.83–0.96) in assignment of which hospitalizations were “for COVID-19” versus “with incidental infection” and that the CDC definition underestimated Direct COVID-19 hospitalizations by 9.8%^11^. Recognizing that some hospitalizations for non-COVID conditions (for example, thromboembolic events, heart failure or COPD exacerbations, diabetic ketoacidosis) may be attributable to the additional physiological burden of concomitant SARS-CoV-2 infection, we classified these hospitalizations as "Potential Contributing" rather than definitively assigning them to the “Direct” versus “Incidental” categories. We believe our 3 tier classification provides more granular information on prognosis and resource utilization in hospitalized patients with SARS-CoV-2 infection. While some may argue that patients admitted for anxiety, depression, or overdoses/suicide attempts within 14 days of a positive test for SARS-CoV-2 infection should not be included in the “Potential Contributing” category, we decided to do so as it was impossible to judge to what extent testing positive contributed to these patients’ mental health and we wanted to be as conservative as possible in declaring hospitalizations unrelated to COVID-19. It should be noted that this was a small number of patients (78) and if we classified these in the “Incidental” category instead it would have had minimal impact on our findings, increasing the prevalence of hospitalizations with incidental SARS-CoV-2 infections from 26.0 to 26.6%.

## Conclusion

Patients admitted to hospital with a SARS-CoV-2 infection who met our definition of “Incidental” have increased proportionately over time but, contrary to assumptions, these patients still have substantial health resource needs. Thus, policy decision-making should not solely focus on the number of hospitalizations in which COVID-19 is implicated as the direct cause, but, using standardized definitions, should include all hospitalizations in patients with SARS-CoV-2 infection, which in turn may facilitate more accurate resource planning for hospitals. In addition, there is a need for standardized definitions to modify the existing ICD-10 code U07.1 to allow the distinction between those hospitalizations where COVID-19 is the Direct cause, a Potential Contributing Factor, or an Incidental finding to refine healthcare administrative data commonly used to inform hospital capacity planning and public health measures.

## Supplementary Information

Supplementary Information.

## Data Availability

To comply with Canada’s Healthcare Privacy Legislation, the dataset used for this study cannot be made publicly available. The dataset from this study is held securely in coded form within the University of British Columbia office of the Canadian COVID-19 Emergency Department Rapid Response Network (CCEDRRN, see www.ccedrrn.com). While legal data sharing agreements between the investigators, participating institutions, and CCEDRRN prohibit us from making the dataset publicly available, access may be granted to those who meet pre-specified criteria for confidential access, and requests should be forwarded to admin.ccedrrn@ubc.ca.
