# Supplementary material for: The burden of incidental SARS-CoV-2 infections in hospitalized patients across pandemic waves in Canada
Source: Sci Rep. 2023 Apr 24;13:6635. doi: 10.1038/s41598-023-33569-2 (PMC10123574; doi:10.1038/s41598-023-33569-2)
Supplement: Supplementary file 1 — Supplementary Information. [file 41598_2023_33569_MOESM1_ESM.docx]

e Appendix for: **THE BURDEN OF INCIDENTAL SARS-CoV-2 INFECTIONS IN HOSPITALIZED PATIENTS ACROSS PANDEMIC WAVES IN CANADA**

Finlay A. McAlister,^1,2^ MD MSc

Jeffrey P. Hau,^3^ MSc

Clare Atzema,^4,5^ MD MSc

Andrew D. McRae,^6^ MD PhD

Laurie J. Morrison,^4^ MD MSc
Lars Grant,^7,8^ MD PhD

Ivy Cheng,^4^ MD PhD

Rhonda J. Rosychuk^9^, PhD

Corinne M. Hohl,^3,10^ MD MHSc

For the [Canadian COVID-19 Emergency Department Rapid Response Network](https://www.ccedrrn.com/) (CCEDRRN) Investigators

**eFigure 1.** Proportion admitted where COVID-19 was the direct cause of admission across pandemic period by province

**eTable 1: Most Responsible Diagnoses at time of hospital discharge**

|  | **COVID-19 is direct cause of hospitalization**  **(n=9,942)** | **COVID-19 is a potential contributing factor for hospitalization**  **(n=630)** | **SARS-CoV-2 infection is incidental and not related to cause of hospitalization**  **(n=3,718)** | **All SARS-CoV-2 infected Patients**  **(n=14,290)** |
| --- | --- | --- | --- | --- |
| COVID-19 or viral pneumonia or pneumonia or ARDS or respiratory failure or influenza-like illness or bronchitis or multi-organ failure | 9,841 (99.0) | - | - | 9,841 (68.9) |
| Sepsis | 88 (0.9) | - | - | 88 (0.6) |
| Upper respiratory tract infection or pharyngitis or sinusitis | 13 (0.1) | - | - | 13 (0.1) |
| Diseases of the nervous system | - | 164 (26.0) | 396 (10.7) | 560 (3.9) |
| Diseases of the circulatory system | - | 86 (13.7) | 431 (11.6) | 517 (3.6) |
| Injury or poisoning  -overdose or suicide attempt  -fall-related injury  -non-fall related injury (MVA, etc)  -accidental poisoning or drug interactions | - | 107 (17.0)  39  68  0  0 | 245 (6.6)  0  0  61  184 | 352 (2.5) |
| Mental and behavioural disorders  -anxiety  -depression  -other (bipolar, schizophrenia, etc) | - | 40 (6.4)  8  32  0 | 218 (5.9)  0  0  218 | 258 (1.8) |
| Endocrine, nutritional and metabolic diseases | - | 96 (15.2) | 106 (2.9) | 202 (1.4) |
| Diseases of the genitourinary system | - | 38 (6.0) | 144 (3.9) | 182 (1.3) |
| Diseases of the digestive system | - | suppressed | 574 (15.4) | 575 (4.0) |
| Diseases of the musculoskeletal system and connective tissue | - | suppressed | 306 (8.2) | 308 (2.2) |
| Infectious and parasitic diseases (not including COVID-19, pneumonia, viral pneumonia, upper respiratory tract infection, pharyngitis, sinusitis, or sepsis) | - | suppressed | 266 (7.2) | 268 (1.9) |
| Neoplasms | - | suppressed | 125 (3.4) | 126 (0.9) |
| Diseases of the skin and subcutaneous tissue | - | suppressed | 102 (2.7) | 103 (0.7) |
| Diseases of the blood and blood-forming organs and certain disorders involving the immune mechanism | - | - | 85 (2.3) | 85 (0.6) |
| Diseases of the respiratory system (other than COVID-19, pneumonia, viral pneumonia, bronchitis) | - | - | 83 (2.2) | 83 (0.6) |
| Factors influencing health status and contact with health services | - | - | 64 (1.7) | 64 (0.5) |
| Pregnancy, childbirth and the puerperium | - | - | 28 (0.8) | 28 (0.2) |
| Symptoms, signs and abnormal clinical and laboratory findings | - | - | 7 (0.2) | 7 (0.1) |
| Diseases of the eye | - | - | suppressed | suppressed |
| Other | - | 92 (14.6) | 535 (14.4) | 627 (4.4) |

Results in cells with less than 5 individuals suppressed for privacy; MVA = motor vehicle accidents
